# Social and Demographic Determinants of Health: A Descriptive Study on the Impact of Place of Residence and Community Belonging

**DOI:** 10.3390/healthcare13101125

**Published:** 2025-05-12

**Authors:** Roser Pedret-Llaberia, Teresa Basora-Gallisà, Sara Martínez-Torres, Sergi Rodríguez-Soler, Meritxell Pallejà-Millán, Agata Buscemi, Cristina Rey-Reñones, Francisco M. Martín-Luján

**Affiliations:** 1 Department of Primary Care Camp de Tarragona, Primary Care Center Mont-roig, Catalan Healthcare Institute, 43300 Mont-roig, Spain; 2 ISAC Research Group (Health Interventions and Community Activities, 2021 SGR 00884), Foundation University Institute for Research in Primary Health Care—IDIAPJGol, 08007 Barcelona, Spain; 3 Primary Healthcare Research Support Unit Camp de Tarragona, Jordi Gol University Institute for Primary Care Research (IDIAP Jordi Gol), 53-55 Cami de Riudoms, 43202 Reus, Spain; 4 Faculty of Psychology and Education Sciences, Universitat Oberta de Catalunya (UOC), 08018 Barcelona, Spain; 5 School of Medicine and Health Sciences, Universitat Rovira I Virgili, 43201 Reus, Spain; 6 School of Architecture, Universitat Rovira I Virgili, 43204 Reus, Spain

**Keywords:** primary healthcare, community-based participatory research, social determinants of health

## Abstract

Background: Social conditions in which individuals live, work, and interact have a significant impact on health. Extensive evidence suggests that place of residence influences health disparities and overall well-being. Understanding the characteristics of a population can help shape healthcare policies that contribute to improved public well-being. Objective: The aim of this research was to describe the main characteristics of the population under study, considering place of residence and other sociodemographic factors. Methods: This is a descriptive study. A tailored 79-item questionnaire was developed based on validated instruments, including variables related to sociodemographic, physical activity, rest and sleep patterns, emotional well-being, and sense of community belonging. The sample was obtained through an open invitation to the general population, ensuring representativeness in terms of sex, age, and nationality. Data were analysed using standard statistical methods for this type of study. Results: A total of 487 different response profiles were collected, representing 3.7% of the total population. Not all participants answered every question. Overall, 33.6% of respondents reported having a chronic disease, with the highest prevalence among individuals over 65 years old. Notably, those who live alone are not necessarily the ones who report feeling the loneliest. The findings highlight the need for new social and healthcare policies at the institutional level. Conclusions: No statistically significant differences were found based on place of residence, except for those related to physical activity and sense of community belonging.

## 1. Introduction

Although great importance has traditionally been placed on genetic and biological factors, an increasing body of evidence highlights the significant impact of social conditions, such as where people live, work, and interact, on health. Therefore, there is growing recognition of the need to address social inequalities through a sensitive approach to the social determinants of health, incorporating concepts like health assets and salutogenesis. Salutogenesis is understood as a commitment to generating health in people’s lives rather than merely addressing the cure of diseases [1,2].

A recent systematic review suggests that salutogenic interventions could improve key factors such as sense of coherence, quality of life, self-efficacy, self-management, sense of purpose, and mental health in older adults [3].

There is extensive evidence that place of residence contributes to disparities in health and its quality. More recent studies confirm that residential location significantly influences both health and mortality rates [4].

A recent study on geographic disparities in mortality in the U.S. highlights that these disparities have been increasing, with significant differences across states and regions. The main disparities are influenced by factors such as income, access to healthcare, and lifestyle habits. While income accounted for only 3% of mortality inequalities in 1992, by 2016, this figure had risen to 58%. Additionally, metropolitan coastal areas have experienced a greater increase in life expectancy compared to rural areas, where improvements have been more limited. Regarding specific diseases, disparities in lung cancer and respiratory disease mortality have increased, particularly among older women. Furthermore, this study emphasizes that birthplace is a stronger predictor of mortality than place of residence, underscoring the lasting impact of early-life exposures [5].

Generally, research indicates that improving health status leads to reduced healthcare costs. While aging has traditionally been considered the most important determining factor, studies now show that healthy lifestyle and socioeconomic status—such as education and marital status—are more influential than other biological factors [6].

In the region of Tarragona (Catalonia, Spain) lies the municipality of Mont-roig del Camp (41°05′18″ N 0°57′28″ E), with a population of approximately 14,000 inhabitants and an area of 63.31 km^2^. The municipality is divided by various roads (highways, expressways, and national roads), creating distinct urban centres within the population [7].

The main objective of this study is to identify the key characteristics of the population, with particular attention paid to their place of residence and other sociodemographic factors.

## 2. Materials and Methods

### 2.1. Study Design

This is a cross-sectional descriptive study about the key sociodemographic characteristics of the population under study.

### 2.2. Setting and Study Period

This study was conducted within the context of primary and community healthcare, led by the primary care team of the basic health area. Data collection took place between February and May 2024.

### 2.3. Population and Sample

The study population consists of residents of a municipality with approximately 14,000 inhabitants, distributed across several urban centres. The prominent coastal urban centre has over 7000 residents (Mont-roig del Camp), while a second urban centre, located about 15 km inland (Miami Playa), has more than 4000 residents. Additionally, there are other urban centres, referred to as “urbanizations,” with lower population densities.

To calculate the sample size, data from the website of the Catalan Institute of Statistics (IDESCAT) as of 2022 [8] were used, with a reference population of 13,101 residents. An α risk of 0.9 was accepted for a precision of +/− 0.05 units, and it was determined that a sample of 266 subjects was necessary for this level of precision. The ARCSINUS approximation (GRANMO Sample Size Calculator v.8 available at https://www.datarus.eu/aplicaciones/granmo/ (accessed on 1 March 2021)) was used.

### 2.4. Data Collection Method and Tools

To develop the questionnaire, investigators followed a series of methodological steps to ensure its validity and reliability. The selection of items was based on an analysis of the existing literature on the subject. Previous studies, validated questionnaires from similar research, and relevant theoretical frameworks were reviewed. The thematic blocks were defined based on a literature review that identified key dimensions related to the study’s objective. Several indicators were established considering the reviewed questionnaires, including the following: (a) physical and relaxing activities, health, and well-being; (b) community; (c) relationships; (d) perception; (e) integration and belongingness; (f) respondents in paid work; (g) personal functioning; (h) idleness and physical activity; (i) territory; and (j) communication.

The final questionnaire was developed after an extensive bibliographic search conducted by a team of experts in the field [9,10,11,12,13,14,15]. From each article, the most relevant aspects of the concept of well-being were identified and analysed, including the methodologies employed, the contextual conditions under which the studies were developed, and the objectives pursued. The questions were grouped according to the indicators they addressed, thus creating different indicators that we found interesting to evaluate. The interviews conducted within the community constitute a fundamental empirical basis for generating indicators that are representative of its socio-territorial reality. Through qualitative and quantitative questions, these surveys translate perceptions, everyday practices, and interpersonal relationships into observable variables that reflect the community’s characteristics and overall well-being. The questions were organized into four macro-categories: (a) the community; (b) the environment; (c) well-being; and (d) intra-community relations. Subsequently, specific categories were defined, such as community, physical activity, social relationships, integration and attachment, perception, cohesion, and social capital. These categories enable the derivation of indicators with both social and territorial validity. The questionnaire design was based on a rigorous process of review, expert consultation, and pilot testing, ensuring its validity and reliability for data collection in the proposed study. This entire process was carried out by a team of experts in the field.

Finally, a 79-item ad hoc questionnaire was created. The questionnaire was anonymous and designed to be self-administered through a QR code. Assistance was also offered to individuals with limited access to the technology. For the analysis in this study, some of the questions were selected and grouped into the five blocks of interest of (a) questions on sociodemographic variables; (b) questions on physical exercise variables; (c) questions on sleep and rest variables; (d) questions on emotional state variables; and (e) questions on community belonging variables:

(a) Sociodemographic variables included age (12–17, 18–30, 31–45, 46–65, 66–75, and over 75); gender (man, woman, non-binary, or other); current status (student, active worker, retired, unemployed, disabled, or home/family caregiver); usual residence (Mont-roig del Camp, Miami Playa, or urbanizations); country of origin (Spanish, Moroccan, Romanian, German, French, Italian, Russian, Algerian, Belgian, English, Colombian, or other); and “Who do you live with?” (multiple choice: family, friends, co-workers, partner, alone, or other). Participants were asked if they had any chronic diseases.
(b) Variables related to physical exercise included questions about whether they engage in physical activity, relaxing activities, and the types of activities (walking, hiking, cycling, running, swimming, and dancing).
(c) Variables related to rest and sleep included the number of hours of sleep and sleep quality (poor, fair, good, or very good).
(d) Variables related to emotional state included questions about happiness (rated from 1 to 10). Participants were asked, “What activities have helped improve your well-being?” (social activities, cultural activities, and emotional activities). They had to respond with “yes or no” to the following questions: “Do you feel lonely?”, “Do you feel that your life is worthwhile?”, “Do you feel a sense of accomplishment from what you do?”, and “Do you find it hard to return to normal if things go wrong?”.
(e) Variables related to the sense of community belonging included “Do you feel part of a community?” and “Do you participate in organizing community activities?”. They were also asked if they use any digital tools for health or well-being.

The sample was obtained by inviting people to participate in the survey at the health centre or at community activities organized or led by health professionals. Healthcare professionals invited the public to participate by posting posters in frequently visited public places, such as sports centres, schools, pharmacies, and recreational and leisure areas. These posters contained a QR code that linked to an online questionnaire. To facilitate the public’s completion for the questionnaire, some healthcare professionals and administrators from the healthcare centre volunteered to help answer the questions for those seeking assistance or experiencing difficulties. Participants were recruited at the two health centres in the city. At the beginning of the questionnaire, only those residing in the town were asked to respond.

All residents aged 12 and over who agreed to participate were included in the study. Those under 16 years of age were asked to provide parental consent. Those unable to communicate in one of the following languages were excluded from the study: Spanish, Catalan, English, or French.

To obtain a representative sample, the invitation to participate was targeted based on the population’s distribution by gender, age (0–14 years, 15–64 years, 65–84 years, and over 85 years), and nationality (Spanish and foreign).

### 2.5. Statistical Analysis

A descriptive analysis of categorical and quantitative variables was conducted. Categorical variables were described by their frequency distribution, and continuous variables were described by the mean and standard deviation (SD) or median and interquartile range (IQR), depending on whether or not they had a normal distribution, respectively. The normality of the variables was assessed using the Kolmogorov test.

Age-related data were categorized into three groups: under 30 years, between 31 and 65 years, and over 65 years. Nationality was grouped into Spanish, European, North African, or other.

A bivariate analysis was performed using the paired Student *t* test to compare pre-training and post-training quantitative variables or the Mann–Whitney U test when appropriate. The McNemar test was used for categorical variables.

All analyses were carried out using the statistical package R (R Foundation for Statistical Computing, Vienna, Austria, 2024), version 4.4.2. The level of significance was set at *p* < 0.05.

### 2.6. Ethical Approval

The COMONT study received approval from the Ethics Committee for Clinical Research of the IDIAP Jordi Gol Foundation on 27 April 2022 (22/103-P). Data confidentiality is protected under the Spanish law governing the protection of personal data (Ley Orgánica de Protección de Datos de Carácter Personal y garantía de los derechos digitales; 03/2018, 5 December). Participation in the study was voluntary, and only participants who signed an informed consent form were included in this study.

## 3. Results

Table 1 presents the distribution of the total population of Mont-roig del Camp in 2022, which consists of 13,101 individuals, categorized by gender, age groups, and nationality. It also includes the estimated sample size of 266 individuals required to obtain a representative sample for each subgroup. A total of 487 different response profiles were obtained (3.7% of the total population). The number of responses exceeds the estimated sample size in all categories.

Regarding gender, the population is almost evenly distributed between men, who represent 51.4%, and women, who make up 48.6%. The estimated sample followed this proportion, but the actual number of responses was significantly higher than expected, especially among women, where the response rate reached 207.31% of the estimated sample.

In terms of age groups, the majority of the population falls within the 15 to 64 years category, a trend that is reflected in both the estimation and the responses obtained. Response rates exceeded in all age groups, with the highest over-representation occurring among individuals aged 85 and over, whose response rate reached 222.7% of the estimated sample.

Regarding nationality, most of the population is Spanish, representing 73.8%, while foreign nationals account for 26.2%. The estimation sought to maintain this proportion, but the actual responses showed a higher participation of Spanish nationals, reaching 170.1% of the estimated sample.

Overall, the final number of responses collected was higher than expected across all demographic segments, ensuring a robust and diverse dataset for analysis.

### 3.1. Main Characteristics of the Overall Population

Table 2 presents a detailed sociodemographic and health-related analysis of a sample of 395 individuals from Mont-roig del Camp, Miami Playa, and surrounding urbanizations. It includes information on gender distribution, age groups, activity status, nationality, chronic diseases, physical exercise, sleep patterns, emotional well-being, and community engagement. The data are stratified by geographic location to compare differences among the three areas. Notably, there were no statistically significant differences in the main variables studied, except for physical exercise practice and the sense of belonging to the community. Not all questions were answered by all participants.

#### 3.1.1. Sociodemographic Variables

Regarding sociodemographic variables, no statistically significant differences were found based on place of residence. When analysing age groups (<30 years, 30–65 years, and >65 years), no differences were observed in terms of gender or place of residence among respondents. However, a higher proportion of younger individuals was found in Miami Playa (57.5%) compared to Mont-roig del Camp (24.1%) and urbanizations (18.4%).

According to the nationality, the population over 65 years old was predominantly Spanish (83.8%), compared to 78.3% of those under 30 years old, with no statistically significant differences. In terms of cohabitation, 86.5% of individuals over 65 years old did not live with family (*p* < 0.001), compared to 17.2% of those under 30 years old. Living alone was more common among those over 65 years old (21.2%) compared to 3.1% of those under 30 and 11% of those aged 30 to 65 (*p* = 0.009). Additionally, older adults were the least likely to use digital tools, with 69% reporting usage, compared to 83.3% of those under 30 and 90.3% of those aged 30–65 (*p* = 0.007).

A total of 33,6% of the surveyed population reported having a chronic disease, with the highest prevalence among individuals over 65 years old (63.3%), compared to 38.2% in the 30–65 age group and 5.8% among those under 30 (*p* < 0.001).

When analysing nationality by place of residence, Miami Playa exhibited a more diverse population, with 23.8% of respondents being foreign nationals, compared to 11.9% in Mont-roig del Camp and 19.4% in urbanizations (*p* = 0.003).

#### 3.1.2. Physical Exercise Variables

When examining physical exercise by age group, walking was the most frequently reported activity among individuals over 65 years old (73.9%), compared to 41.8% of younger participants and 63.8% of those aged 30–65 (*p* = 0.002). In contrast, running was more common among younger individuals, with 21.8% engaging in this activity (*p* = 0.005).

In terms of physical activity by place of residence, the most sedentary population was found in coastal areas, where 41.3% of respondents reported not engaging in any physical or relaxing activity. Running was most frequently reported among inland residents (22.1%), compared to 10.2% in coastal areas and 8.16% in residential developments (*p* = 0.03).

#### 3.1.3. Rest and Sleep Variables

Individuals over 65 years old reported fewer hours of sleep on average (6.45 h, SD = 1.67) compared to younger participants (*p* = 0.002). However, no statistically significant differences were found between sleep variables and place of residence.

#### 3.1.4. Emotional State Variables

Happiness scores were highest among individuals over 65 years old, with an average of 7.96 out of 10 (SD = 1.72) (*p* = 0.012). Similarly, this group reported the highest sense of success in life, with 81.2% responding affirmatively, compared to 62% of those under 30 and 57.9% of those aged 30–65 (*p* = 0.018).

There were no statistically significant differences between loneliness and age. However, loneliness was more prevalent among individuals who identified as female and was significantly higher among non-binary individuals, with three out of five respondents in this group reporting feelings of loneliness (*p* = 0.01). Regarding employment status, the highest prevalence of loneliness was observed among unemployed individuals (44.82%), followed by those identifying as disabled (29%), compared to students (21.5%), employed individuals (20.23%), and retirees or homemakers (12.5%) (*p* = 0.01). Additionally, individuals of foreign origin reported higher levels of loneliness than those identifying as Spanish (32.83% vs. 18.79%; *p* = 0.01). Conversely, no significant differences were found regarding place of residence and participation in or organization of community activities. Likewise, there were no significant associations between loneliness and morbidity-related factors.

Regarding feelings of loneliness, living alone was not necessarily associated with increased loneliness. Specifically, 83.9% of individuals who reported feeling lonely did not live alone, while only 16.1% of those living alone reported experiencing loneliness. No statistically significant differences were found between loneliness and living alone.

Sleep quality was notably worse among those who reported feeling lonely. Specifically, 25% of individuals experiencing loneliness rated their sleep as very poor, compared to 9% of those who did not feel lonely. Additionally, 49.29% of lonely individuals rated their sleep as fair, compared to 39.62% of those not experiencing loneliness, while 19.7% of the lonely group reported good sleep quality compared to 41.13% of the non-lonely group. Only 5.6% of lonely individuals described their sleep as very good, compared to 10% of those not experiencing loneliness (*p* < 0.001).

#### 3.1.5. Community Belonging Variables

Regarding community participation, the results showed that women participated more frequently, regardless of age group. When assessing community involvement, inland residents reported organizing community activities more frequently (26.4%) compared to 16.6% of coastal residents and 10.9% of those living in residential developments (*p* = 0.04). This group also reported higher participation in community activities.

Additionally, Spanish nationals were significantly more likely to participate in community activities (31.53%) compared to individuals of other nationalities (10%) (*p* = 0.003). No significant differences were found regarding other family or cohabitation characteristics.

## 4. Discussion

The results of this study provide a detailed description of the main characteristics of a population divided across multiple urban areas, considering additional variables of interest such as age.

Approximately 4% of the population responded to the questionnaire. While other studies suggest that a response rate of 40–50% is necessary for a sample to be considered representative, the method of cluster selection based on age, gender, and origin ensures that the responses obtained are still representative of the study population. According to a recent systematic review, the average sample size for interviews was 1422 ± 261, with 279 ± 111 for face-to-face interviews (48,700/13). It is also important to note that no incentive was provided to encourage participation, despite evidence suggesting that prepaid incentives can increase response rates in health surveys [16]. The questionnaires were mainly self-administered through an electronic link and data collection website, and other studies have shown that web-based questionnaires are viable for data collection in population-based epidemiological studies without significant differences compared to paper-based questionnaires [17]. Consequently, the findings of this study confirm an equitable distribution of both sexes and various age groups.

The study’s findings reveal that there are no significant differences according to place of residence for the main characteristics studied, except for nationality, where coastal residents were found to have a more diverse population. However, no statistically significant differences were observed when grouping by region (Europeans, North Americans, South Americans, or others). Notably, the sense of community belonging was distinctly higher among inland residents compared to those living on the coast or in urbanizations.

The sense of community belonging is an important factor influencing both psychological and physical well-being. This study may serve as a precursor for the development of new health policies and strategies. According to the literature, the sense of community belonging varies based on various demographic, geographic, and social factors, as seen in this study. Community belonging has been associated with self-reported health outcomes, even after controlling for socioeconomic status, chronic diseases, and other health-related variables [18,19].

Regarding chronic diseases, 33.6% of the surveyed population reported having one, with the prevalence rising to 63.3% among individuals over 65 years old. These figures are significantly lower than those reported by North American studies, where 51.8% of adults reported having a chronic disease, and 27.2% had multiple chronic conditions [20]. Other European studies estimate the prevalence of chronic diseases in the adult population at 21.43% [21]. In nearby populations, such as in Catalonia, a recent study found that 46.6% of the population had at least one chronic disease, which is slightly higher than the prevalence observed in this study but still comparable to other communities.

Another noteworthy result of this study is the percentage of the population living alone, which is higher among those over 65 years old. A recent systematic review and meta-analysis published in 2022 indicates that loneliness varies worldwide and within Europe, with lower rates in Northern European countries and higher rates in Eastern European countries. That study reports that the prevalence of loneliness in Spain is around 9%, similar to the 11.5% in our study, and 21.2% among those over 65, which aligns with rates found in Eastern European countries [22]. It is significant that those who live alone are not the ones who feel lonelier, which invites new socio-health strategies for public institutions.

Although there is some controversy regarding the scales and indices used to measure happiness [23], a recent study showed that the happiness score of European populations places Finland at the highest level with a score of 7.81, slightly higher than the 7.3 (SD = 1.9) observed in our study. This score is comparable to countries like Switzerland and Sweden [24].

Regarding sleep hours, it is well known that sleep deprivation negatively impacts cognitive performance, mood, and metabolic variables. Chronic sleep deprivation can have serious long-term health effects [25]. Recent studies in China and Canada report average sleep durations of 6.82 h and 7.12 h per day, respectively, with the population in our study falling below both [26,27]. According to the updated recommendations from the National Sleep Foundation, adults aged 65 and over should sleep between 7 and 8 h per day [28].

The use of digital health tools varies widely depending on the population and context. A North American study found that 49.2% of the population reported seeking health information online, and 18.5% had at least one technology-based interaction with the healthcare system [29]. In our study, 38.7% of respondents reported using a health app, with higher usage among younger populations, as suggested by most of the literature [30].

### Limitations

This study has several limitations that should be considered when interpreting the results. First, the use of self-reported measures may introduce bias, as participants could have over- or under-reported certain behaviours or experiences. Second, the response rate of 3.7% may affect the representativeness of the sample, potentially introducing selection bias, as individuals more engaged in community activities or those that are health conscious may have been more likely to participate. Additionally, while the study provides valuable insights into the population, the generalizability of the findings may be limited by the sample size and the specific context of the Spanish municipality. Future research could address these limitations by incorporating longitudinal designs to assess causality and exploring interventions aimed at enhancing community belonging and engagement.

## 5. Conclusions

This study offers a comprehensive analysis of the main characteristics of a population across urban areas, considering key sociodemographic variables such as age, gender, and origin. The findings demonstrate that, in general, place of residence does not significantly affect the studied characteristics, with the exception of the sense of community belonging, which was notably higher among inland residents. The prevalence of chronic diseases in this population was lower than that reported in other international studies, although it increased significantly with age. Interestingly, loneliness was more common among those aged 65 and above, but living alone did not correlate directly with higher feelings of loneliness, indicating a need for targeted social and health interventions. Furthermore, the use of health apps and sleep patterns were consistent with global trends. These results underscore the importance of integrating social and community factors into public health policy to address the evolving needs of diverse populations.

## Figures and Tables

**Table 1:** Total of responses according to estimated subcategories.

					Percentage of Responses in Relation to the Estimation
			Total Mont-Roig del Camp Population (2022 Year) N = 13,101 (%)	Estimation N = 266	Total Responses (%)
					N = 487 (159.0)
Gender		Man	6734 (51.4)	136.7	150 (109.7)
		Woman	6367 (48.6)	129.3	268 (207.3)
Age-groups ^1^	0 to 14 years	Man	1016 (7.8)	20.6	52 (133.0)
		Woman	910 (7.0)	18.5	
	15 to 64 years	Man	4482 (34.2)	91.0	300 (170.0)
		Woman	4212 (32.2)	85.5	
	65 to 84 years	Man	1140 (8.70)	23.2	50 (111.6)
		Woman	1067 (8.1)	21.7	
	85 and more	Man	96 (0.7)	2.0	20 (222.7)
		Woman	178 (2.6)	7.0	
Nationality	Spanish	Man	4963 (37.9)	100.8	334 (170.1)
		Woman	4707 (35.9)	95.6	
	Foreign	Man	1771 (13.5)	36.0	79 (113.4)
		Woman	1660 (12.7)	33.7	

^1^ Age groups were categorized as under 30 years, between 31 and 65 years, and over 65 years, following the classification provided by IDESCAT.

**Table 2:** Sociodemographic, health, and well-being characteristics of the surveyed population by place of residence.

		All N = 395 (%)	Mont-roig del Camp	Miami Playa	Urbanizations	*p*-Value
Sociodemographic variables						
Gender		N = 389				
	Man	141 (36.2)	40 (31.7)	72 (36.5)	29 (43.9)	0.588
	Woman	243 (62.5)	85 (67.5)	121 (61.4)	37 (56.1)	
	Not binary	5 (1.3)	1 (0.8)	4 (2)		
Age groups		N = 390				
	<30 age	87 (22.3)	21 (16.7)	50 (25.4)	16 (23.9)	0.286
	30–65 age	234 (60)	82 (65.1)	116 (58.9)	36 (53.7)	
	>65 age	69 (17.7)	23 (18.3)	31 (15.7)	15 (22.4)	
Activity						
		N = 393				
	Student	60 (15.3)	9 (7.1)	39 (19.6)	12 (17.9)	0.393
	Active Worker	194 (49.4)	80 (63)	83 (41.7)	31 (46.3)	
	Retired	77 (19.6)	27 (21.3)	35 (17.6)	15 (22.4)	
	Unemployed	33 (8.4)	5 (3.9)	23 (11.6)	5 (7.5)	
	Home/family caregiver	13 (3.3)	3 (2.4)	8 (4)	2 (3)	
	Disabled	16 (4.1)	(2.4)	11 (5.5)	2 (3)	
Nationality						
		N = 395				
	Spanish	319 (80.8)	111 (88.1)	154 (76.2)	54 (80.6)	0.288
	European	32 (8.1)	6 (4.8)	21 (10.4)	5 (7.5)	
	North African	12 (3)	3 (2.4)	8 (4)	1 (1.5)	
	South American	3 (0.8)	1 (0.8)	2 (1)	0	
	Others	29 (7.34)	5 (4)	17 (8.4)	7 (10.4)	
Chronic disease (yes)	N = 332	216 (65.1)	73 (71.6)	106 (61.3)	37 (64.9)	0.224
Physical exercise						
Physical activity (yes)	N = 386	241 (60.7)	77 (60.6)	115 (56.6)	49 (73.1)	0.026
Rest and sleep						
Sleep hours *	N = 330	6.54 (1.3)	6.75 (1.3)	6.43 (1.4)	6.48 (1.1)	0.114
Sleep quality		N = 326				
	Poor	43 (13.2)	8 (8)	26 (15.4)	9 (15.8)	0.463
	Fair	136 (41.7)	39 (39)	71 (42)	26 (45.6)	
	Good	117 (35.9)	43 (43)	57 (33.7)	17 (29.8)	
	Very good	30 (9.2)	10 (10)	15 (8.9)	5 (8.8)	
Emotional state						
Happiness *	N = 369	7.35 (1.9)	7.62 (1.74)	7.15 (2.02)	7.4 (1.78)	0.105
Feels lonely (yes)	N = 356	78 (21.9)	20 (17.4)	44 (24.6)	14 (22.6)	0.344
Believes their life has value (yes)	N = 201	171 (85.1)	53 (89.8)	84 (82.4)	34 (85)	0.439
Feels successful in life (yes)	N= 196	124 (63.3)	38 (66.7)	59 (59.6)	27 (67.5)	0.558
Can return to normality (yes)	N = 196	88 (44.9)	20 (33.9)	49 (50.5)	19 (47.5)	0.121
Community						
Feels part of a community (yes)	N = 302	186 (61.6)	65 (71.4)	96 (61.5)	25 (45.5)	<0.001
Uses a health app	N = 186	72 (38.7)	21 (39.6)	34 (35.4)	17 (45.9)	0.529

* Data is expressed as mean and SD.

## Data Availability

Data are contained within the article.
